# Genetic risk variants for brain disorders are enriched in cortical H3K27ac domains

**DOI:** 10.1186/s13041-019-0429-4

**Published:** 2019-01-28

**Authors:** Eilis Hannon, Sarah J. Marzi, Leonard S. Schalkwyk, Jonathan Mill

**Affiliations:** 1 University of Exeter Medical School, RILD Building, Royal Devon & Exeter Hospital, University of Exeter, Barrack Rd, Exeter, EX2 5DW UK; 2 Blizard Institute, Queen Mary University of London, London, E1 2AD UK; 3 School of Biological Sciences, University of Essex, Colchester, CO4 3SQ UK

**Keywords:** H3K27ac, Active enhancer, Promoter, GWAS, Brain disorder, LD score regression, Psychiatric illness, Neurodegenerative disease, Epigenetics

## Abstract

Most variants associated with complex phenotypes in genome-wide association studies (GWAS) do not directly index coding changes affecting protein structure. Instead they are hypothesized to influence gene regulation, with common variants associated with disease being enriched in regulatory domains including enhancers and regions of open chromatin. There is interest, therefore, in using epigenomic annotation data to identify the specific regulatory mechanisms involved and prioritize risk variants. We quantified lysine H3K27 acetylation (H3K27ac) - a robust mark of active enhancers and promoters that is strongly correlated with gene expression and transcription factor binding – across the genome in entorhinal cortex samples using chromatin immunoprecipitation followed by highly parallel sequencing (ChIP-seq). H3K27ac peaks were called using high quality reads combined across all samples and formed the basis of partitioned heritability analysis using LD score regression along with publicly-available GWAS results for seven psychiatric and neurodegenerative traits. Heritability for all seven brain traits was significantly enriched in these H3K27ac peaks (enrichment ranging from 1.09–2.13) compared to regions of the genome containing other active regulatory and functional elements across multiple cell types and tissues. The strongest enrichments were for amyotrophic lateral sclerosis (ALS) (enrichment = 2.19; 95% CI = 2.12–2.27), autism (enrichment = 2.11; 95% CI = 2.05–2.16) and major depressive disorder (enrichment = 2.04; 95% CI = 1.92–2.16). Much lower enrichments were observed for 14 non-brain disorders, although we identified enrichment in cortical H3K27ac domains for body mass index (enrichment = 1.16; 95% CI = 1.13–1.19), ever smoked (enrichment = 2.07; 95% CI = 2.04–2.10), HDL (enrichment = 1.53; 95% CI = 1.45–1.62) and trigylcerides (enrichment = 1.33; 95% CI = 1.24–1.42). These results indicate that risk alleles for brain disorders are preferentially located in regions of regulatory/enhancer function in the cortex, further supporting the hypothesis that genetic variants for these phenotypes influence gene regulation in the brain.

**Electronic supplementary material:**

The online version of this article (10.1186/s13041-019-0429-4) contains supplementary material, which is available to authorized users.

## Main text

There has been major progress in identifying genetic risk variants for complex brain traits including neurodegenerative diseases (for example Alzheimer’s disease and amyotrophic lateral sclerosis [1–3]) and neuropsychiatric illnesses (for example schizophrenia and major depressive disorder [4–7]). A key challenge is to understand the biological effects of these genetic risk factors, especially because the actual gene(s) involved in mediating phenotypic variation are not necessarily the closest to the most significant genetic variant in genome-wide association studies (GWAS). The majority of GWAS variants do not directly index or tag coding changes affecting protein structure. Instead, common variants associated with disease are preferentially located in regulatory domains such as active enhancers and regions of open chromatin [8, 9], and therefore are hypothesized to act by influencing gene regulation [10]. There is, therefore, much interest in using epigenomic data to improve our understanding of how genetic variants associated with complex disease mediate differences in gene activity and regulation. Given the tissue-specific nature of gene regulation, it is critical these relationships are explored in relevant tissues; existing epigenomic annotation data has been largely generated in easily accessible tissues and cells, or commercially available cell lines. In particular, datasets based on the human brain are lacking, limiting the downstream interpretation of GWAS findings for brain traits. Recently, we quantified genome-wide patterns of lysine H3K27 acetylation (H3K27ac) - a robust mark of active enhancers and promoters that is strongly correlated with gene expression and transcription factor binding – using ChIP-seq in an extensive collection of entorhinal cortex samples (*n* = 47) [11]. In this study, we used these data to perform enrichment analyses of GWAS variants for a range of brain traits (attention-deficit hyperactivity disorder (ADHD), Alzheimer’s disease, autism, amyotrophic lateral sclerosis (ALS), major depressive disorder, bipolar disorder and schizophrenia) using linkage disequilibrium (LD) score regression [12] to test the hypothesis that the majority of these variants act by influencing gene regulation in the brain.

Detailed methods on the experimental procedures and informatics pipeline used to derive the set of cortical H3K27ac peaks have been previously described [11]. Briefly, post-mortem entorhinal cortex samples from 47 donors were provided by the MRC London Neurodegenerative Disease Brain Bank (https://www.kcl.ac.uk/ioppn/depts/bcn/index.aspx). The entorhinal cortex, which is located in the medial temporal lobe, has an important role in memory formation and has been implicated in a range of neuropsychiatric and neurological phenotypes [13]. We annotated genome-wide patterns of H3K27ac in the entorhinal cortex using chromatin immunoprecipitation (ChIP) followed by highly parallel sequencing (ChIP-seq). After stringent quality control of the raw H3K27ac ChIP-seq data, we obtained a mean of 30,032,623 (SD = 10,638,091) sequencing reads per sample, representing the most extensive analysis of H3K27ac in the human entorhinal cortex yet undertaken. H3K27ac peaks were called from the combined set of high quality mapped reads across all samples using *MACS2* [14], and filtered to exclude those located on sex chromosomes, in unmapped contigs and mitochondrial DNA. In total, we generated a final dataset of 178,454 autosomal entorhinal cortex H3K27ac peaks which were used in the analyses presented here.

To test for enrichment of GWAS variants in H3K27ac peaks from adult cortex, we performed partitioned heritability analysis using the LD score regression software (https://github.com/bulik/ldsc) [12, 15]. Briefly, this method assumes that the test statistic for a given genetic variant also captures the effect of all other variants in LD with it; the number of additional variants tagged by the particular variant under consideration is measured by its ‘LD score’. Genuine polygenic effects are present, therefore, if the test statistics positively correlate with the LD scores. The method can be applied either across the genome to derive an estimate of total heritability or to subsets of genetic variants annotated to genomic features, so called partitioned heritability. Enrichment is determined if there is a stronger, positive correlation between the test statistics and LD scores for variants within a category relative to other categories. LD scores were generated based on custom annotations derived from our H3K27ac peaks and 1000 genomes reference data (downloaded alongside the software from https://data.broadinstitute.org/alkesgroup/LDSCORE/). The baseline model proposed by Finucane et al. [15] - based on the union of non-specific functional annotation categories including coding, UTR, promoters, introns, histone marks (H3K4me1, H3K4me3, H3K9ac5, H3K27ac), DNase I hypersensitivity site (DHS) regions, chromHMM/Segway predictions of underlying chromatin states derived from ENCODE annotations, regions that are conserved in mammals, super-enhancers and active enhancers - was taken as the background for enrichment testing. Genetic variants were annotated to two non-overlapping categories defined as follows: 1) entorhinal cortex H3K27ac peaks and 2) any other functional annotation category included in the baseline model. Heritability statistics for each annotation category were then calculated using publicly available GWAS results for seven psychiatric and neurodegenerative traits (ADHD [16], Alzheimer’s disease [1], autism [17], amyotrophic lateral sclerosis (ALS) [2], major depressive disorder [7], bipolar disorder [5] and schizophrenia [4, 6, 18]) and 14 non-brain phenotypes (birth length [19], body mass index (BMI) [20, 21], height [21, 22], cigarettes per day [23], ever smoked [23], coronary artery disease [24], Crohn’s disease [25], inflammatory bowel disease [25], ulcerative colitis [25], high density lipoprotein (HDL) [26], low density lipoprotein (LDL) [26], total cholesterol [26], triglycerides [26] and type 2 diabetes [27]) (See Additional file 1: Table S1). Enrichment statistics for each GWAS trait were calculated as the proportion of heritability attributed to that category divided by the proportion of SNPs annotated to that category, with 95% confidence intervals used to identify significant enrichment statistics. These represent the enrichment relative to the set of more broadly defined functional elements derived from cross-tissue datasets included in the baseline model.

**Fig. 1:**
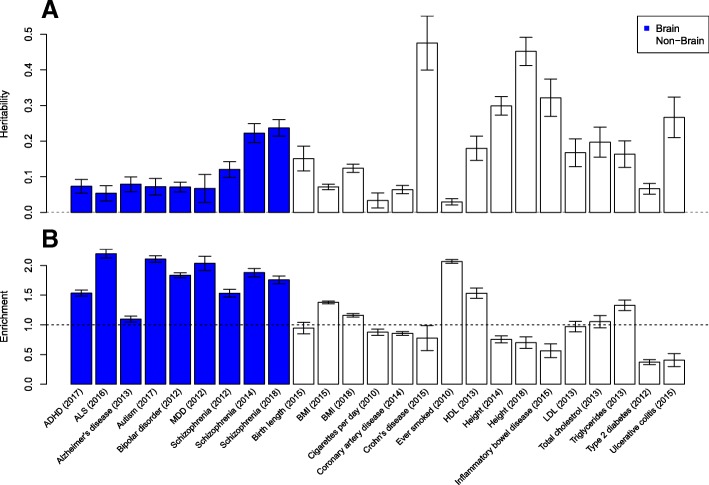
Enrichment of heritability within entorhinal cortex H3H27ac peaks. **a** Bar plot of total heritability estimates calculated across genetic variants located within any functional element. **b** Bar plot of cortical H3K27ac enrichment statistics. Enrichment was calculated as the proportion of heritability divided by the proportion of variants within autosomal H3K27ac peaks in the entorhinal cortex, relative to values for the set of more broadly defined functional elements derived from cross-tissue datasets. Error bars represent 95% confidence intervals; dashed horizontal lines indicate null values

We first estimated the total heritability of each trait using variants annotated to any functional genomic annotation category to confirm that the included GWAS had sufficient power to quantify heritability with enough precision to permit downstream enrichment analyses. Across the seven brain traits, the total heritability estimates ranged from 0.0535 for ALS (95% confidence interval (0.0321, 0.0749)) to 0.237 for schizophrenia (95% confidence interval (0.214, 0.260)) (Fig. 1a). Next, we estimated the partitioned heritability attributable to variants located within entorhinal cortex H3K27ac peaks. This ranged from 0.0302 for Alzheimer’s disease (95% confidence interval (0.013, 0.0478)) to 0.146 for schizophrenia (95% confidence interval (0.121, 0.170)); all seven brain traits had significantly non-zero estimates of heritability within H3K27ac peaks (Table 1). Finally, we compared partitioned heritability estimates between entorhinal cortex H3K27ac peaks and more broadly defined functionally active regions of the genome identified across multiple cell types. For all seven brain traits, heritability was enriched within the entorhinal cortex H3K27ac peaks (Fig. 1b). The strongest enrichment was for ALS (enrichment = 2.20; 95% confidence interval (2.12, 2.27)), followed by autism (enrichment = 2.11; 95% confidence interval (2.05, 2.16)) and major depressive disorder (enrichment = 2.04; 95% confidence interval (1.92, 2.16)); the lowest enrichment was for Alzheimer’s disease (enrichment = 1.10; 95% confidence interval (1.05, 1.15). Enrichments for all seven brain traits remained significant when correcting for the number of independent tests performed (Additional file 2: Table S2). We next compared these results to those for the 14 non-brain phenotypes; although most were found to have non-zero heritability estimates for variants located within entorhinal cortex H3K27ac peaks, these were generally not enriched relative to functional elements defined across multiple tissue types. The exceptions were for body mass index (BMI) (enrichment = 1.16; 95% confidence interval (1.13, 1.19)), ever smoked (enrichment = 2.07; 95% confidence interval 2.04, 2.10), high density lipoprotein (HDL) (enrichment = 1.53; 95% confidence interval (1.45, 1.62)) and triglycerides (enrichment = 1.33; 95% confidence interval = (1.24, 1.42)). These results are interesting given that both BMI and smoking are known to have a neurobiological component, and it is plausible that genetic variation associated with these traits may have mechanistic effects in the cortex.

**Table 1:** Enrichment of heritability within entorhinal cortex H3K27ac peaks

Trait (date GWAS published)	Total observed heritability				Partitioned heritability for EC H3K27ac peaks				Enrichment			
	Estimate	SE	95% CI Lower	95% CI Upper	Estimate	SE	95% CI Lower	95% CI Upper	Estimate	SE	95% CI Lower	95% CI Upper
A												
ADHD (2017)	0.073	0.010	0.054	0.092	0.039	0.009	0.021	0.057	1.534	0.026	1.483	1.585
ALS (2016)	0.054	0.011	0.032	0.075	0.041	0.013	0.015	0.067	2.199	0.038	2.125	2.274
Alzheimer’s disease (2013)	0.079	0.011	0.058	0.099	0.030	0.009	0.013	0.048	1.097	0.026	1.047	1.148
Autism (2017)	0.072	0.012	0.049	0.095	0.053	0.010	0.034	0.072	2.108	0.027	2.055	2.162
Bipolar disorder (2012)	0.071	0.007	0.057	0.085	0.046	0.008	0.031	0.060	1.836	0.022	1.793	1.879
MDD (2012)	0.067	0.020	0.028	0.107	0.048	0.021	0.006	0.090	2.036	0.061	1.916	2.155
Schizophrenia (2012)	0.120	0.011	0.098	0.142	0.064	0.012	0.041	0.088	1.533	0.034	1.466	1.600
Schizophrenia (2014)	0.222	0.014	0.196	0.249	0.146	0.013	0.121	0.170	1.879	0.036	1.809	1.950
Schizophrenia (2018)	0.237	0.012	0.214	0.260	0.145	0.012	0.122	0.168	1.758	0.034	1.692	1.824
B												
Birth length (2015)	0.151	0.018	0.116	0.186	0.050	0.017	0.016	0.084	0.947	0.050	0.850	1.044
BMI (2015)	0.072	0.004	0.064	0.079	0.034	0.004	0.027	0.042	1.378	0.011	1.356	1.401
BMI (2018)	0.124	0.006	0.112	0.136	0.050	0.005	0.040	0.060	1.162	0.015	1.132	1.192
Cigarettes per day (2010)	0.033	0.011	0.012	0.054	0.010	0.009	−0.008	0.028	0.876	0.026	0.825	0.928
Coronary artery disease (2014)	0.064	0.006	0.052	0.075	0.019	0.006	0.008	0.030	0.855	0.016	0.823	0.887
Crohn’s disease (2015)	0.475	0.039	0.399	0.551	0.129	0.038	0.055	0.202	0.776	0.108	0.564	0.987
Ever smoked (2010)	0.029	0.005	0.020	0.038	0.021	0.006	0.010	0.032	2.067	0.016	2.035	2.099
HDL (2013)	0.180	0.018	0.146	0.214	0.096	0.015	0.066	0.126	1.533	0.044	1.447	1.619
Height (2014)	0.299	0.013	0.273	0.325	0.079	0.010	0.058	0.099	0.754	0.030	0.696	0.813
Height (2018)	0.452	0.020	0.412	0.492	0.110	0.017	0.077	0.144	0.701	0.049	0.604	0.797
Inflammatory bowel disease (2015)	0.322	0.027	0.269	0.374	0.063	0.021	0.023	0.103	0.564	0.059	0.449	0.679
LDL (2013)	0.167	0.020	0.128	0.206	0.057	0.016	0.026	0.087	0.971	0.044	0.884	1.058
Total cholestrol (2013)	0.197	0.021	0.155	0.239	0.072	0.018	0.036	0.108	1.053	0.053	0.950	1.157
Triglycerides (2013)	0.163	0.019	0.126	0.200	0.076	0.015	0.045	0.106	1.329	0.044	1.242	1.416
Type 2 diabetes (2012)	0.066	0.008	0.051	0.081	0.009	0.007	−0.006	0.023	0.372	0.021	0.330	0.413
Ulcerative colitis (2015)	0.267	0.029	0.209	0.324	0.038	0.020	−0.001	0.076	0.406	0.056	0.296	0.516

Heritability and enrichment statistics from partitioned heritability analysis performed using the LD score regression software for A) brain traits and B) non-brain traits. EC = entorhinal cortex

In summary, we report an enrichment of heritability within active regions of regulatory and enhancer function in the adult entorhinal cortex for seven brain disorders. This augments an existing body of evidence that genetic variants identified in GWAS are involved in gene regulation [10]. Furthermore, it uses regulatory domains defined in the relevant tissue and demonstrates that these regions are more informative than functional elements defined across a panel of tissues and cell types, highlighting the importance of generating cell-type and tissue-specific epigenomic annotation datasets. Although our data represents the largest entorhinal cortex H3K27ac dataset generated to date, we were restricted to performing a global enrichment analysis. Future analyses in larger numbers of samples should aim to undertake a genetic analysis of each peak and align these results with GWAS results in order to identify the specific peaks, and ultimately genes, associated with genetic variants identified in genetic studies of brain traits. There are a number of limitations to our study. First, although one of the strengths of our study is the use of cortical H3K27ac data, our ChIP-seq analyses were performed on bulk tissue and future studies should aim to generate epigenomic annotation data for specific neural cell-types [28]. Second, we have only considered one specific epigenetic mark, H3K27ac; future studies exploring a more comprehensive set of marks may yield insights into the exact mechanism by which genetic variants influence gene regulation. Third, the H3K27ac data were generated in elderly adult post-mortem brain, which may be less relevant for neurodevelopmental brain phenotypes such as autism, ADHD and schizophrenia. In conclusion, our results support the hypothesis that genetic variants associated with brain disorders exert their effect through gene regulation in the brain. Future studies should aim to identify the specific regulatory elements affected by genetic variants associated with brain disorders and the genes that are transcriptionally altered by these differences.

## Additional files

Additional file 1: **Table S1.** Details of the GWAS datasets used in this study. (PDF 46 kb)

Additional file 2: **Table S2.** Enrichments for all seven brain traits remained significant when correcting for the number of independent tests performed. (PDF 30 kb)

## Abbreviations

ADHD: Attention deficit hyperactivity disorder
ALS: Amyotrophic lateral sclerosis
BMI: Body mass index
ChIP: Chromatin immunoprecipitation
DHS: DNase I hypersensitivity site
GWAS: Genome-wide association study
H3K27ac: lysine H3K27 acetylation
HDL: High density lipoprotein
LD: Linkage disequilibrium
LDL: Low density lipoprotein
