# Supplementary material for: Genetic risk variants for brain disorders are enriched in cortical H3K27ac domains
Source: Mol Brain. 2019 Jan 28;12:7. doi: 10.1186/s13041-019-0429-4 (PMC6348673; doi:10.1186/s13041-019-0429-4)
Supplement: Supplementary file 1 — Table S1. Details of the GWAS datasets used in this study. (PDF 46 kb) [file 13041_2019_429_MOESM1_ESM.pdf]

Disease / Trait	Reference	Sample Size	Number of significant loci / SNPs	Primary cohort ethnicity
ADHD	Demontis D, Walters RK, Martin J, Mattheisen M, Als TD, Agerbo E, et al. Discovery Of The First Genome-Wide Significant Risk Loci For ADHD. Nat. Genet. 2018 (15)	20,183 cases and 35,191 controls	304 SNPs (12 loci)	Danish, European, North American, Chinese
Alzheimer's Disease	Lambert JC, Ibrahim-Verbaas CA, Harold D, Naj AC, Sims R, Bellenguez C, et al. Meta-analysis of 74,046 individuals identifies 11 new susceptibility loci for Alzheimer's disease. Nat Genet. 2013;45(12):1452-8 (1)	17,008 cases and 37,154 controls;	20 loci	European Ancestry
Autism	Grove J, Ripke S, Als TD, Mattheisen M, Walters R, Won H, et al. Common risk variants identified in autism spectrum disorder. bioRxiv. 2017 (16)	18,381 cases and 27969 controls	3 loci	Danish, European
Amyotrophic lateral sclerosis (ALS)	van Rheenen W, Shatunov A, Dekker AM, McLaughlin RL, Diekstra FP, Pulit SL, et al. Genome-wide association analyses identify new risk variants and the genetic architecture of amyotrophic lateral sclerosis. Nat Genet. 2016 (2)	12,577 cases and 23,475 controls	7 loci	European Ancestry
Major depressive disorder	Wray NR, Ripke S, Mattheisen M, Trzaskowski M, Byrne EM, Abdellaoui A, et al. Genome-wide association analyses identify 44 risk variants and refine the genetic architecture of major depression. Nat Genet. 2018;50(5):668-81. (7)	135,458 cases and 344,901 controls	44 loci	European Ancestry
Bipolar disorder	Psychiatric GWAS Consortium Bipolar Disorder Working Group. Large-scale genome-wide association analysis	7,481 cases and	4 loci	German, European-American, British,

	of bipolar disorder identifies a new susceptibility locus near ODZ4. Nat Genet. 2011;43(10):977-83. (5)	9,250 controls		Canadian, Scottish, Norwegian, Irish, British
Schizophrenia	Schizophrenia Working Group of the PGC, Ripke S, Neale B, Corvin A, Walters J, Farh K, et al. Biological insights from 108 schizophrenia-associated genetic loci. Nature. 2014;511(7510):421-+. (4)	36,989 cases and 113,075 controls	108 loci	European, East Asian
	Pardiñas AF, Holmans P, Pocklington AJ, Escott-Price V, Ripke S, Carrera N, et al. Common schizophrenia alleles are enriched in mutation-intolerant genes and in regions under strong background selection. Nat Genet. 2018;50(3):381-9. (6)	11,260 cases and 24,542 controls	179 SNPS (145 loci)	European Ancestry
	Schizophrenia Working Group of the PGC, Ripke S, Sanders A, Kendler K, Levinson D, Sklar P, et al. Genome-wide association study identifies five new schizophrenia loci. Nat Genet. 2011;43(10):969-U77. (17)	9,394 cases and 12,462 controls	7 loci	European Ancestry (UK, USA, UK, Bulgaria, Ireland, Portugal, Sweden, Germany, Denmark, Norway, The Netherlands)
Birth length	van der Valk RJ, Kreiner-Møller E, Kooijman MN, Guxens M, Stergiakouli E, Sääf A, et al. A novel common variant in DCST2 is associated with length in early life and height in adulthood. Hum Mol Genet. 2015;24(4):1155-68. (18)	28,459	7 SNPs	Multiple
Body mass index (BMI)	Locke AE, Kahali B, Berndt SI, Justice AE, Pers TH, Day FR, et al. Genetic studies of body mass index yield new insights for obesity biology. Nature. 2015;518(7538):197-206. (19)	322,154	77 loci	European Ancestry
	Yengo L, Sidorenko J, Kemper KE, Zheng Z, Wood AR, Weedon MN, et al. Meta-analysis of genome-wide association studies for height and body mass index in ~700,000 individuals of European ancestry. bioRxiv. 2018. (20)	681,275	716 SNPs (416 loci)	European Ancestry

Height	Yengo L, Sidorenko J, Kemper KE, Zheng Z, Wood AR, Weedon MN, et al. Meta-analysis of genome-wide association studies for height and body mass index in ~700,000 individuals of European ancestry. bioRxiv. 2018. (20)	693,529	3,290 SNPs (712 loci)	European Ancestry
	Wood AR, Esko T, Yang J, Vedantam S, Pers TH, Gustafsson S, et al. Defining the role of common variation in the genomic and biological architecture of adult human height. Nat Genet. 2014;46(11):1173-86. (21)	253,288	423 loci	European Ancestry
Cigarettes per day	Tobacco and Genetics Consortium. Genome-wide meta-analyses identify multiple loci associated with smoking behavior. Nat Genet. 2010;42(5):441-7. (22)	74,053	3 loci	European Ancestry
Ever smoked	Tobacco and Genetics Consortium. Genome-wide meta-analyses identify multiple loci associated with smoking behavior. Nat Genet. 2010;42(5):441-7. (22)	74,053	8 SNPs	European Ancestry
Coronory Heart Disease	CARDIoGRAMplusC4D Consortium, Nikpay M, Goel A, Won HH, Hall LM, Willenborg C, et al. A comprehensive 1,000 Genomes-based genome-wide association meta-analysis of coronary artery disease. Nat Genet. 2015;47(10):1121-30. (23)	60,801 cases and 123,504 controls	55 loci	European Ancestry, Indian, Pakistaini, Chinese, Korean, Hispanic African American
Crohn's disease	Liu JZ, van Sommeren S, Huang H, Ng SC, Alberts R, Takahashi A, et al. Association analyses identify 38 susceptibility loci for inflammatory bowel disease and highlight shared genetic risk across populations. Nat Genet. 2015;47(9):979-86. (24)	5956 cases and 14927 controls	38 loci	European Ancestry
IBD	Liu JZ, van Sommeren S, Huang H, Ng SC, Alberts R, Takahashi A, et al. Association analyses identify 38 susceptibility loci for inflammatory bowel disease and highlight shared genetic risk across populations. Nat Genet. 2015;47(9):979-86. (24)	12882 cases 21770 controls		European Ancestry

Ulcerative colitis	Liu JZ, van Sommeren S, Huang H, Ng SC, Alberts R, Takahashi A, et al. Association analyses identify 38 susceptibility loci for inflammatory bowel disease and highlight shared genetic risk across populations. Nat Genet. 2015;47(9):979-86. (24)	6968 cases and 20464 controls		European Ancestry and non-European (including East Asian, South Asian, African)
High Density Lipoprotein (HDL)	Global Lipids Genetics Consortium, Willer CJ, Schmidt EM, Sengupta S, Peloso GM, Gustafsson S, et al. Discovery and refinement of loci associated with lipid levels. Nat Genet. 2013;45(11):1274-83. (25)	188,577	60 loci	European Ancestry and non-European (including East Asian, South Asian, African)
Low Density Lipoprotein (LDL)	Global Lipids Genetics Consortium, Willer CJ, Schmidt EM, Sengupta S, Peloso GM, Gustafsson S, et al. Discovery and refinement of loci associated with lipid levels. Nat Genet. 2013;45(11):1274-83. (25)		30 loci	European Ancestry and non-European (including East Asian, South Asian, African)
Total Cholesterol	Global Lipids Genetics Consortium, Willer CJ, Schmidt EM, Sengupta S, Peloso GM, Gustafsson S, et al. Discovery and refinement of loci associated with lipid levels. Nat Genet. 2013;45(11):1274-83. (25)		49 loci	European Ancestry and non-European (including East Asian, South Asian, African)
Triclycerides	Global Lipids Genetics Consortium, Willer CJ, Schmidt EM, Sengupta S, Peloso GM, Gustafsson S, et al. Discovery and refinement of loci associated with lipid levels. Nat Genet. 2013;45(11):1274-83. (25)		28 loci	European Ancestry and non-European (including East Asian, South Asian, African)
Type 2 diabetes	Morris AP, Voight BF, Teslovich TM, Ferreira T, Segre AV, Steinthorsdottir V, et al. Large-scale association analysis provides insights into the genetic architecture and pathophysiology of type 2 diabetes. Nat Genet. 2012;44(9):981-+. (26)	12,171 cases and 56,862 controls	65 loci	European Ancestry

SUPPLEMENTARY TABLE 1. Details of the GWAS datasets used in this study.
