# Supplementary material for: Genetic risk variants for brain disorders are enriched in cortical H3K27ac domains
Source: Mol Brain. 2019 Jan 28;12:7. doi: 10.1186/s13041-019-0429-4 (PMC6348673; doi:10.1186/s13041-019-0429-4)
Supplement: Supplementary file 2 — Table S2. Enrichments for all seven brain traits remained significant when correcting for the number of independent tests performed. (PDF 30 kb) [file 13041_2019_429_MOESM2_ESM.pdf]

GWAS trait	Category	Enrichment	SE	No Correction		Correcting for 7 brain traits		Correcting for all 21 traits	
				95% CI Lower	95% CI Upper	99.3% CI Lower	99.3% CI Upper	99.64% CI Lower	99.64% CI Upper
**ADHD (2017)**	**Brain**	1.534	0.026	1.483	1.585	1.465	1.603	1.456	1.612
**ALS (2016)**	**Brain**	2.199	0.038	2.125	2.274	2.097	2.302	2.083	2.315
**Alzheimer's disease (2013)**	**Brain**	1.097	0.026	1.047	1.148	1.028	1.167	1.019	1.176
**Autism (2017)**	**Brain**	2.108	0.027	2.055	2.162	2.035	2.181	2.025	2.191
**Bipolar disorder (2012)**	**Brain**	1.836	0.022	1.793	1.879	1.778	1.895	1.770	1.902
**MDD (2012)**	**Brain**	2.036	0.061	1.916	2.155	1.871	2.200	1.850	2.221
**Schizophrenia (2012)**	**Brain**	1.533	0.034	1.466	1.600	1.441	1.625	1.429	1.637
**Schizophrenia (2018)**	**Brain**	1.758	0.034	1.692	1.824	1.668	1.848	1.656	1.860
**Birth Length (2015)**	**Non-brain**	0.947	0.050	0.850	1.044	0.813	1.080	0.796	1.098
**BMI (2015)**	**Non-brain**	1.378	0.011	1.356	1.401	1.347	1.409	1.343	1.413
**Cigarettes per day (2010)**	**Non-brain**	0.876	0.026	0.825	0.928	0.805	0.947	0.796	0.957
**Coronary artery disease (2014)**	**Non-brain**	0.855	0.016	0.823	0.887	0.811	0.899	0.805	0.905
**Crohn's Disease (2015)**	**Non-brain**	0.776	0.108	0.564	0.987	0.486	1.066	0.448	1.104
**Ever Smoked (2010)**	**Non-brain**	2.067	0.016	2.035	2.099	2.023	2.111	2.018	2.117
**HDL (2013)**	**Non-brain**	1.533	0.044	1.447	1.619	1.415	1.651	1.400	1.667
**Height (2014)**	**Non-brain**	0.754	0.030	0.696	0.813	0.674	0.835	0.664	0.845
**Inflamatory bowel disease (2015)**	**Non-brain**	0.564	0.059	0.449	0.679	0.406	0.722	0.385	0.743
**LDL (2013)**	**Non-brain**	0.971	0.044	0.884	1.058	0.852	1.091	0.836	1.106

**Total cholestrol (2013)**	**Non-brain**	1.053	0.053	0.950	1.157	0.911	1.195	0.893	1.214
**Triglycerides (2013)**	**Non-brain**	1.329	0.044	1.242	1.416	1.210	1.448	1.195	1.463
**Type 2 diabetes (2012)**	**Non-brain**	0.372	0.021	0.330	0.413	0.315	0.429	0.307	0.436
**Ulceritive colitus (2015)**	**Non-brain**	0.406	0.056	0.296	0.516	0.255	0.557	0.235	0.577

SUPPLEMENTARY TABLE 2. Enrichments for all seven brain traits remained significant when correcting for the number of independent tests performed.
